# Deep Multimodal Detection in Reduced Visibility Using Thermal Depth Estimation for Autonomous Driving

**DOI:** 10.3390/s22145084

**Published:** 2022-07-06

**Authors:** Sungan Yoon, Jeongho Cho

**Affiliations:** Department of Electrical Engineering, Soonchunhyang University, Asan 31538, Korea; ysa258@sch.ac.kr

**Keywords:** object detection, image dehazing, depth estimation, autonomous driving

## Abstract

Recently, the rapid development of convolutional neural networks (CNN) has consistently improved object detection performance using CNN and has naturally been implemented in autonomous driving due to its operational potential in real-time. Detecting moving targets to realize autonomous driving is an essential task for the safety of drivers and pedestrians, and CNN-based moving target detectors have shown stable performance in fair weather. However, there is a considerable drop in detection performance during poor weather conditions like hazy or foggy situations due to particles in the atmosphere. To ensure stable moving object detection, an image restoration process with haze removal must be accompanied. Therefore, this paper proposes an image dehazing network that estimates the current weather conditions and removes haze using the haze level to improve the detection performance under poor weather conditions due to haze and low visibility. Combined with the thermal image, the restored image is assigned to the two You Only Look Once (YOLO) object detectors, respectively, which detect moving targets independently and improve object detection performance using late fusion. The proposed model showed improved dehazing performance compared with the existing image dehazing models and has proved that images taken under foggy conditions, the poorest weather for autonomous driving, can be restored to normal images. Through the fusion of the RGB image restored by the proposed image dehazing network with thermal images, the proposed model improved the detection accuracy by up to 22% or above in a dense haze environment like fog compared with models using existing image dehazing techniques.

## 1. Introduction

The rapid development of artificial intelligence technology based on convolutional neural networks (CNN) has considerably expanded the applicable fields. Subsequently, there has been widespread interest in deep learning-based object detection algorithms that apply to autonomous driving. According to the object detection methods, deep learning-based object detection algorithms are divided into 1-stage and 2-stage detectors. 1-stage detectors, which include You Only Look Once (YOLO) [1], Single Shot Multi-Box Detector (SSD) [2], and RetinaNet [3], guarantee rapid execution speed by performing object categorization and extracting the bounding box, which shows the object locations simultaneously. Meanwhile, 2-stage detectors, such as Regions with CNN (R-CNN) [4], Fast R-CNN [5], and Faster R-CNN [6], offer high accuracy by the first searching areas where objects are to be found and then categorizing objects according to where they were discovered. While the 1-stage detectors offer high potential in real-time due to rapid execution speed, they have lower accuracy than 2-stage detectors. The 2-stage detectors offer higher accuracy, but their real-time operation is mostly impossible due to slow execution speed. Because of these differences between the detectors, object detection models are applied in various fields based on the purpose of usage. Therefore, developing a model that offers both high accuracy and real-time detection concurrently remains of interest, especially in autonomous driving [7,8].

The main role of autonomous driving is to accurately and rapidly detect vehicles, pedestrians, traffic signals and signs, and other objects surrounding vehicles to guarantee driving safety. Object detection is a key part of autonomous driving since, for vehicles to drive safely at a high speed, they must accurately detect all objects on the road in real-time. Thus, it plays an important role in monitoring traffic, preventing collision accidents, avoiding obstacles, etc. [9,10]. While RGB camera-based object detection models in previous studies [11,12] offer stable and fast-moving target detection performance, they are prone to the turbid medium like haze or fog in the atmosphere, which expresses objects in the image in the achromatic color instead of their natural color, leading to increased image ambiguity. Particularly, haze or fog is a frequently observed weather condition caused by particles formed by clustered vapors in the air, which further scatter light, reducing the contrast and decreasing the saturation of the image. Such a dim image obscures the boundary between an object and its background, and makes the object almost invisible, drastically reducing detection performance. Therefore, to secure stable object detection performance, much focus has been on effective and reliable methods for restoring these obscured images to clear ones by removing external factors like haze [13,14,15].

Generally, image dehazing algorithms can be divided into image enhancement [16,17] and image restoration [18,19] methods. The image enhancement methods are classic image processing techniques, such as histogram equalization, wavelet transformation, luminance, and contrast transformation, among others. These techniques can relatively remove haze with ease, thereby increasing the fidelity of hazy images with low contrast. However, they rely on local contrast distribution information, causing an overall image imbalance and color distortion. The image restoration method, which sets an estimation model for dim images and compensates for distortion by inferring the process in which the image becomes dim, offers more natural and detailed image dehazing results than the aforementioned classic methods. Xie et al. [18] proposed a haze removal method using dark channel prior (DCP), a well-known image dehazing algorithm, and multi-scale retinex (MSR), a popular contrast improvement technique, and extracted a function map similar to a transmission map. Li et al. [19] attempted haze removal by estimating an improved transmission map using a homomorphic filter and an improved DCP. Both [18] and [19] showed good dehazing performance on the images with various fog types by estimating a transmission map using DCP, a method for calculating the airlight and transfer rate using the fact that the minimum value among the RGB values in an area without fog is very small compared with that of a foggy area. However, such methods produce images with low contrast, can produce a halo artifact due to the fog in the border area of the image, and may require a considerable amount of calculation for refining the transfer rate which was estimated in a block shape.

Recently, active research has been conducted on deep learning-based image dehazing for high applicability and effective transmission map estimation for images shot under varying conditions [20,21,22]. Cai et al. [23] removed haze using the atmospheric scattering model estimated with a transmission map obtained by applying dim images to neural networks. Instead of estimating the transmission map and airlight using the reconstructed atmospheric scattering model in previous models, Li et al. [24] proposed a novel cross-sectional design method that directly produces clear images using lightweight CNN. Here, they showed that a high-quality dehazing process can be performed by including it in other deep multi-models. Zhang et al. [25] proposed a method that does not remove haze in an actual image but produces a haze-free image using the generative adversarial network. However, such deep learning-based algorithms have low estimation accuracy due to complicated learning strategies. In addition, they still do not consider depth when estimating the transmission map.

Recently, the moving target detection technique has become a crucial part of autonomous driving, which can guarantee high detection levels due to the rapid development of neural network technologies. However, under poor weather with limited visibility, its moving object detection performance drops considerably. Hence, in this paper, we propose a deep multimodal object detection model with reinforced moving target detection performance under reduced visibility. Its image dehazing uses GoogLeNet, a CNN-based classification model, to learn weather conditions and select an appropriate atmospheric scattering coefficient using the haze level. Furthermore, the proposed model simultaneously performs depth estimation between the object and the camera with Monodepth using the thermal image. Additionally, using the selected atmospheric scattering coefficient and depth information, our proposed model estimates a transmission map and secures a haze-free image using the atmospheric scattering model. Furthermore, it improves estimation performance by detecting objects, using independent YOLO, from clear RGB images with rich color information and thermal images with clear object bounding lines. In addition, it detects an object with the highest probability via late fusion. Overall, the contributions are the following:

1. The study proposed a stable and accurate atmospheric scattering estimation model that independently estimates each parameter of the atmospheric scattering model for image dehazing;
2. We proposed a method for selecting an atmospheric scattering coefficient by estimating the haze level by identifying weather conditions to restore clearer original images since it allows for a more flexible application of the model to various environments;
3. We proposed a novel thermal image-based depth estimation method for removing haze even under poor weather conditions with high dense haze;
4. We proposed a detection model using late fusion of heterogeneous sensors based on dehazed images and demonstrated more improved moving target detection performance;

The rest of this paper is organized as follows: Section 2 describes the image dehazing network with depth estimation. Section 3 explains the object detection framework of YOLO. Section 4 proposes an object detection strategy reinforced by the image dehazing network. Section 5 presents experimental results. Finally, Section 6 presents our conclusions.

## 2. Image Dehazing with Depth Estimation

Haze is the most general phenomenon that obscures visibility and is caused by various particles in the air, such as vapor, dirt, and fog, that scatter atmospheric light. This shortens visibility, obscures images, and reduces image quality. Dim images reduce visibility and significantly weaken the detection performance, thereby causing considerable damage when applied to autonomous driving. The most popular dehazing method removes haze and restores images by estimating atmospheric light and transmission map using an atmospheric scattering model that shows dim images.

### 2.1. Atmospheric Scattering Model

To explain the process of creating hazy images due to atmospheric particles in computer vision, the atmospheric scattering model is defined as follows:

(1) $$I_{h}\left(x\right)=I_{hf}\left(x\right)t\left(x\right)+A\left(1-t\left(x\right)\right)$$

Here, $I_{h}\left(x\right)$ is the observed hazy image, $I_{hf}\left(x\right)$ is the haze-free (or dehazed) image to be restored, $t\left(x\right)$ is a medium transmission map, A is the atmospheric light vector in the RGB domain, and x is the pixel location of the image. The transmission is the part of the light that arrives at the camera without scattering and ranges between 0 and 1. Thus, the objective of dehazing is to restore the haze-free image $I_{hf}\left(x\right)$ from a dim image by estimating $t\left(x\right)$ and A, as shown below.

(2) $$I_{hf}\left(x\right)=\frac{I_{h}\left(x\right)-\bar{A}\left(1-\bar{t}\left(x\right)\right)}{\bar{t}\left(x\right)}$$

Here, $\bar{A}$ is an atmospheric light value arbitrarily estimated between 0 and 255. $\bar{t}\left(x\right)$ is the estimated transmission map, and assuming that atmospheric conditions are even as an exponential function of distance, it depends on two parameters—atmospheric scattering coefficient $\bar{\beta }$ and the depth between the object and the camera $\bar{d}\left(x\right)$. It is expressed as follows:

(3) $$\bar{t}\left(x\right)=e^{-\bar{\beta }*\bar{d}\left(x\right)}$$

Generally, the haze level depends on the number of particles in the atmosphere. $\bar{t}\left(x\right)$ decreases as the distance between the camera and the object increases, signifying an increase in the effect of atmospheric light on the image. Therefore, it causes more light scattering, thereby producing dim objects. As the distance decreases, the atmospheric effect reduces, making the image look relatively clearer.

### 2.2. Image Dehazing Network with Thermal Depth

**Figure 1:**
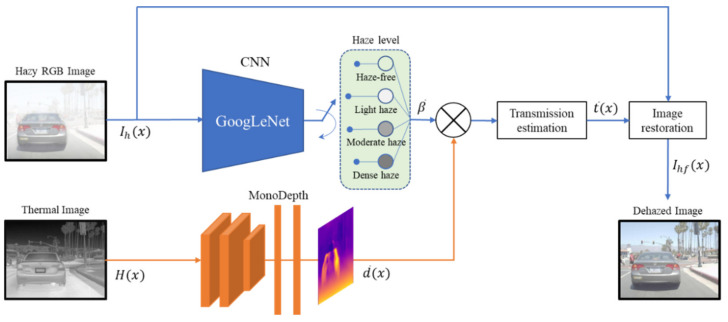
Proposed image dehazing network by incorporating RGB and thermal images.

The dehazing network with RGB and thermal depth (DN-RTD), proposed to remove haze effectively, is designed, as shown in Figure 1, by estimating $\bar{\beta }$, the atmospheric scattering coefficient appropriate to the current atmospheric condition, and $\bar{d}\left(x\right)$, the depth between the object and the camera, using RGB and thermal images.

The proposed dehazing algorithm trains the model using GoogLeNet, a CNN-based classification model, to categorize captured hazy image $I_{h}\left(x\right)$ into four haze levels, namely, haze-free, light haze, moderate haze, and dense hazy, and to select $\bar{\beta }$ corresponding to the classified weather condition. Moreover, the algorithm estimates the depth information $\bar{d}\left(x\right)$ from a thermal image $H\left(x\right)$, not from an RGB image, using Monodepth. After deriving the transmission map $\bar{t}\left(x\right)$, which expresses the level of atmospheric light transmission, from Equation (3) using the above $\bar{\beta }$ and the estimated $\bar{d}\left(x\right)$, the clear image $I_{hf}\left(x\right)$ is extracted via the image restoration process in Equation (2).

The atmospheric scattering coefficient β expresses the degree of light being scattered by particles in the air, and it appears on the overall image rather than a specific area of the image. Therefore, it is estimated by observing the whole image. Authors of [23,24] proposed an estimation method that uses β based on a neural network model, but such a method should estimate an accurate parameter through the training of a neural network model. Therefore, it requires image data at various haze levels and an accurate β, which is labeled according to each data, leading to a high training cost and low estimation accuracy. On this account, for stable β accuracy, we propose a β estimation algorithm that classifies four haze levels using GoogLeNet based on the overall image and selects an appropriate scattering coefficient in a specified scope, rather than estimating an accurate atmospheric scattering coefficient. GoogLeNet is a model that allows deep learning by increasing the length and width of neural networks while maintaining the calculation size using a 1 × 1 convolution-based inception module, average pooling, and auxiliary classifier, among others. It is a CNN model designed for deep learning with small data [26]. As shown in Table 1, for estimating atmospheric scattering coefficient at a specified scope, the training data for GoogLeNet were divided into four categories. The output of the trained GoogLeNet is $G\in \left[1,2,3,4\right]$, denoting the four haze levels representing each atmospheric scattering coefficient corresponding to each state.

### 2.3. Depth Estimation by Thermal Image

The noise level of haze worsens as the atmosphere thickness between the camera and the object increases. As such, the depth information between the camera and the object is a key component in image dehazing algorithms, as well as depth estimation. Generally, depth information can be acquired using light detection and ranging (LiDAR), time-of-flight (ToF) camera, or Kinect depth sensors. However, these are relatively large, expensive, and have a long processing time due to large data sizes. In particular, for a ToF camera, the LED light at a specific wavelength weakens greatly when it is reflected from an object, leading to a limited detection distance. In addition, in outdoor conditions or under strong sunlight, the camera cannot differentiate the LED light and sunlight, making distance detection impossible. To mitigate these issues, some proposed a depth estimation method using a stereo vision sensor, which requires a large calculation to process data and is prone to external noise, such as dirt or lighting [27,28,29]. Godard et al. [30] proposed Monodepth, a model that trains CNN using single images and a disparity map, which shows the difference between two images and extracts depth information from the image reconstruction based on single images. To estimate the mono depth when the left image ($I_{L}$) and the right image ($I_{R}$) exist in pairs, only one image, the left image ($I_{L}$), is input to CNN to extract the left and right disparities ($d_{L}$) and ($d_{R}$), respectively. Then, the training is performed with a loss function, where the reconstructed left image (${\bar{I}}_{L}$) is created by the currently estimated left disparity ($d_{L}$) and right image ($I_{R}$), and the disparity is obtained by the comparison with the existing left image ($I_{L}$). Similarly, the reconstructed right image (${\bar{I}}_{R}$) is created by applying the currently estimated right disparity ($d_{R}$) to the left image ($I_{L}$), and the disparity is obtained by the comparison of the existing right image ($I_{R}$).

The loss function used for Monodepth training from the existing and newly reconstructed image pairs consists of three factors: the similarity between the original image and the reconstructed image ($D_{L,sim})$, the continuity indicating whether the generated image is seamlessly connected ($D_{L,cont}$), and the accuracy of the generated disparity map ($D_{L,acc})$. The first loss function expresses the similarity level of the image and conducts image reconstruction using the left and right disparities acquired from the model. It is defined as follows:

(4) $$D_{L,sim}=\frac{1}{N}\underset{ij}{\sum }\gamma \frac{1-SSIM\left(I_{L}\left(i,j\right),\;\;{\bar{I}}_{L}\left(i,j\right)\right)}{2}+\left(1-\gamma \right)\left|\left|I_{L}\left(i,j\right)-{\bar{I}}_{L}\left(i,j\right)\right|\right|$$

Here, *N* is the total number of pixels in an image, and $\left(i,j\right)$ is the location of the pixel. It is defined by the sum of $SSIM\left(\cdot \right)$ and the L1 regularizer of the two images with a weighting value *γ* set to 0.85 on each. $SSIM\left(\cdot \right)$ is Structural-Similarity-Index-Map, a function that determines the similarity level of images by the luminance, contrast, and structural comparison of the images, in which each function represents the luminance, contrast, and structural comparison, respectively.

(5) $$SSIM\left(\cdot \right)=l\left(i,j\right)\times c\left(i,j\right)\times s\left(i,j\right)$$

The second loss function, shown below, measures gradients in the *x*- and *y*-directions given by $\partial_{x}$ and $\partial_{y}$, among others, so that there is no disconnection in the disparity bounding regions. This has the effect of making the disparities locally smooth by removing the discontinuity of the image of which the depth is not uniform using the gradient of the image.

(6) $$D_{L,cont}=\frac{1}{N}\underset{ij}{\sum }\left|\partial_{x}d_{L}\left(i,j\right)\right|e^{-\left|\left|\partial_{x}I_{L}\left(i,j\right)\right|\right|}+\left|\partial_{y}d_{L}\left(i,j\right)\right|e^{-\left|\left|\partial_{y}I_{L}\left(i,j\right)\right|\right|}$$

Finally, the loss function to make a more accurate disparity map serves to make the left-view disparity map equal to the projected right-view disparity map. To produce a more accurate disparity map, the disparity $d_{L}$ and $d_{R}$ for each of the left and right images are determined, and the difference between them is used for depth estimation in the left-right disparity consistency loss, which is shown below.

(7) $$D_{L,acc}=\frac{1}{N}\underset{ij}{\sum }\left|d_{L}\left(i,j\right)-d_{R+d_{L}\left(i,j\right)}\left(i,j\right)\right|$$

**Figure 2:**
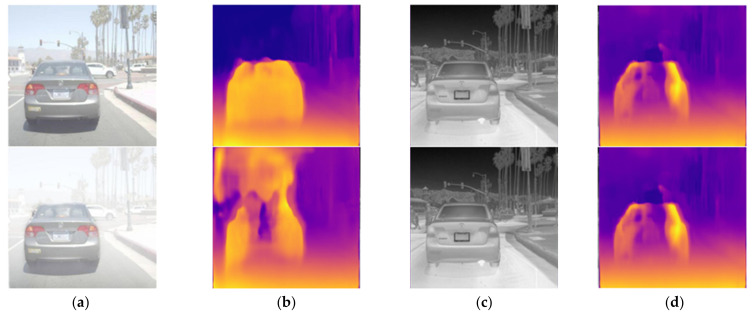
Examples of haze-free (top) and hazy (bottom) images: (**a**) RGB image, (**b**) depth map estimated from the RGB image, (**c**) thermal image, (**d**) depth map estimated from the thermal image.

When estimating a transmission map, depth information is one of the crucial parameters, and acquiring the depth directly from a single image, as shown in Monodepth, saves a considerable cost. However, the RGB camera’s depth estimation accuracy plummets for noise, such as a haze, as shown in Figure 2a,b. Hence, for the low-cost transmission map and accurate estimation, Monodepth is used to estimate stable depth information using thermal images, not RGB images. Thermal images are resistant to haze, and regardless of the haze level, they allow stable estimation of depth information, as shown in Figure 2c,d. Finally, through the above atmospheric scattering coefficient estimated with the neural networks and the depth information estimated with Monodepth, a transmission map is created, and using Equation (2), the original image without haze is restored.

## 3. Object Detection Framework

While object detection technology has advanced considerably due to the development of CNN, the performance improvement of object detection via single sensors is limited and less stable. Therefore, many studies have focused on the development of multi-sensor-based object detection techniques and the improvement of object detection performance through the compensation of multi-sensors. Thus, we proposed to acquire improved object detection performance from the late fusion of the thermal and RBG cameras, which offer clear boundary information of objects and rich color information, respectively.

### 3.1. Real-Time Object Detection

With the various CNN-based object classification models and object recognition algorithms, including GoogLeNet, residual network (ResNet), and visual geometry group from Oxford (VGG), among others, the accuracy of CNN-based object recognition has consistently improved. Object detection, such as the location and identification of an object, is more difficult and has a more complicated structure than simple image recognition, and thus it has not been easily accessed. However, through R-CNN, which uses a CNN-based image classifier, several studies have proposed various object detection models like Fast R-CNN or Faster R-CNN, and improved detection performance. However, these models must calculate the bounding box of the object within the image and class probability separately and conduct object classification via neural networks. This causes considerable training and processing time, thereby making them unsuitable for real-time application. To speed up the object detection speed, the YOLO framework, developed to focus more on real-time object detection, creates the bounding box within the image and class probability as one regressive problem to increase the inference speed, estimates the type and location of an object that was seen before, and trains the neural networks on the whole tasks.

YOLO divides the input image into an S×S grid and predicts the B number of bounding boxes, predetermined in the region where an object is expected to be found using CNN and a confidence score on each bounding box. The bounding box of each region consists of five-dimension vectors $\left(x,y,w,h,C\right)$, where $\left(x,y\right)$ is the central coordinate of the bounding box, $\left(w,h\right)$ is its width and height, and C is the probability that the bounding box is included in a specific class. C is expressed by the multiplication of $\mathrm{Pr}\left(object\right)$, the probability that the object is included, and $IOU^{\begin{array}{c} truth\\ pred \end{array}}$, the width of the IOU (Intersection of Union), where the actual and predicted values overlap, indicating how accurately the bounding box predicted the object.

(8) $$C=\mathrm{Pr}\left(object\right)*IOU^{\begin{array}{c} truth\\ pred \end{array}}$$

Here, if the central coordinates of the predicted bounding box and the ground truth exist in the same region, the bounding box is assumed to include the object, and $\mathrm{Pr}\left(object\right)$ is set to 1; otherwise, it is set to 0. The probability of an object among the N number of classes that could be classified is $\mathrm{Pr}\left(Class_{i}|object\right)$, and the probability that a specific object is included in the predicted bounding box among N classifiable objects is the product of $\mathrm{Pr}\left(Class_{i}|object\right)$ and C as follows:

(9) $$C\mathrm{Pr}\left(Class_{i}|object\right)=\mathrm{Pr}\left(object\right)*IOU^{\begin{array}{c} truth\\ pred \end{array}}*\mathrm{Pr}\left(Class_{i}|object\right)\;=\mathrm{Pr}\left(Class\right)*IOU^{\begin{array}{c} truth\\ pred \end{array}}$$

The bounding box with the highest $C\mathrm{Pr}\left(Class_{i}|object\right)$ among the B numbers of the finally predicted bounding boxes is determined to be the bounding box of the concerned object [1].

### 3.2. Non-Maximum Suppression

Considering only RGB images in object detection under complex conditions like autonomous driving leads to image distortion or damage due to external light like sunlight or lighting and cannot be used at night. To mitigate this issue, additional sensors like LiDAR and Radar have been suggested to be used as multi-sensor fusion methods for the shortcomings of RGB cameras [31]. The early fusion method can completely use the raw data information by fusing pre-processed sensor data. However, this method is sensitive to spatiotemporal data alignment defects among sensors due to correction errors, different sampling frequencies, sensor defects, etc. Nonetheless, the late fusion method offers high flexibility and modularity as it combines the outputs of each network although it requires a slightly higher calculation cost. The mid-fusion method is a compensation method between the early and late fusion methods, which allows the training of various network characteristics. However, finding the optimal fusion method for changing the network structure is challenging.

**Figure 3:**
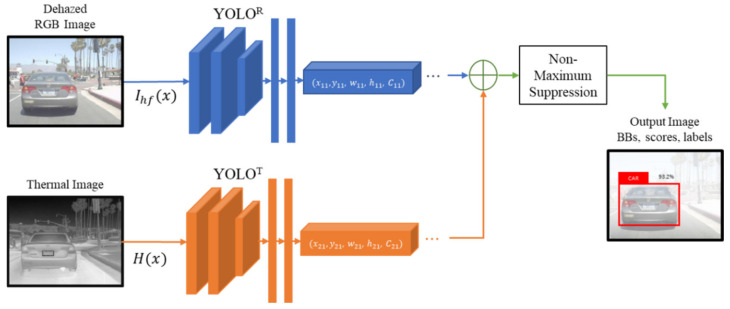
Block diagram of the multimodal YOLO object detection method based on late fusion.

Therefore, this study proposes a multimodal YOLO object detection method using non-maximum suppression (NMS) to efficiently extract the characteristics of an object using rich color and boundary information from RGB and thermal cameras, respectively. Not only does it minimize the intersensory interference through the fusion of the RGB and thermal cameras, but it extracts the optimal object bounding boxes using the late fusion, as shown in Figure 3.

The RGB and thermal images are entered into each YOLO, and the B×2 number of vectors, which shows the location and class of the object, are extracted using NMS and the final bounding box is determined. NMS is connected to the second half of the detection model for improving object detection performance of models like YOLO or SSD to extract the optimal bounding box using the following procedure. First, the bounding box with the highest-class probability against one class is determined and added to the final bounding box list. Second, after comparing the IOU of the selected bounding box and all predicted bounding boxes, if the value is higher than the threshold, the corresponding box is removed. Third, the bounding box with the highest-class probability among the remaining boxes is selected and added to the final bounding box list. After the IOU of the selected bounding box and all remaining bounding boxes are compared, if the value is higher than the threshold, the corresponding box is removed. This process is repeated until no bounding box is left in the list.

## 4. Object Detection in Reduced Visibility

**Figure 4:**
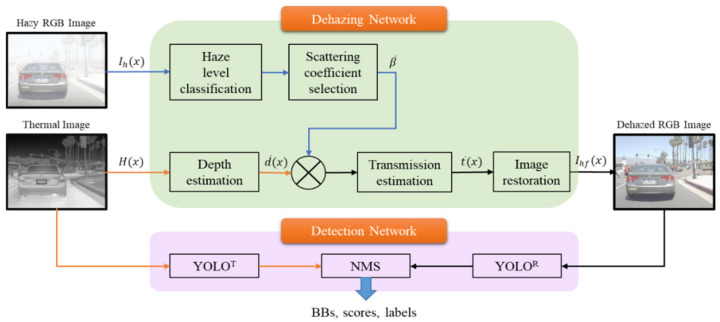
Overview of the improved deep multimodal object detection strategy.

Haze, one of the most frequently observed phenomena in daily life, degrades image quality and weakens detection performance by disturbing the object’s characteristics detection function of a detection model. If a vehicle makes an incorrect control decision when the moving target detection in autonomous driving is disturbed by haze, it can lead to a huge accident. Therefore, to perform stable moving target detection under reduced visibility, this paper proposes an improved deep multimodal YOLO object detection method that performs image dehazing pre-processing using DN-RTD, which is based on the late fusion of the RGB and thermal cameras. The overall block diagram is shown in Figure 4.

The proposed model classifies haze levels into four levels, namely, haze-free, light haze, moderate haze, and dense haze, based on CNN, as the pre-process for extracting clear images under foggy weather conditions. From Table 1, it selects an atmospheric scattering coefficient $\bar{\beta }$ appropriate to each level. Moreover, to estimate stable depth information regardless of the haze level, the model uses thermal image $H\left(x\right)$ and estimates the depth $\bar{d}\left(x\right)$ using Monodepth. Using the derived $\bar{\beta }$ and $\bar{d}\left(x\right)$, the model calculates the transmission map $\bar{t}\left(x\right)$ based on Equation (3) and enters it into the image restoration process in Equation (2) to create a dehazed image $I_{hf}\left(x\right)$. Finally, $I_{hf}\left(x\right)$ and $H\left(x\right)$ are entered into two YOLO models, YOLO^R^ and YOLO^T^, to determine an object based on different images, and NMS is employed to estimate the optimal object detection. Since some haze removal can be performed using the haze level estimates, the proposed model allows an RGB image with improved quality. Additionally, with the late fusion and thermal image, the proposed model can process the rich color and clear boundary information from the RGB and thermal images simultaneously to improve the detection performance in single sensor-based models.

## 5. Experimental Results

The improved deep multimodal object detection model was realized on an NVIDIA GTX 1080ti, Intel Core i7-8700 CPU, and the dataset used for validation was the forward looking infrared thermal dataset [32], containing both RGB and thermal images. The hazed image dataset for the weather classification training for the CNN based on the haze level was generated by acquiring the transmission map using Monodepth and changing the atmospheric scattering coefficient randomly between 0 and 2.3. In addition, the haze removal performance of DN-RTD, the proposed dehazing method, was compared with that of the existing dehazing models using peak signal-to-noise ratio (PSNR) and SSIM.

(10) $$\mathrm{PSNR}=10\log_{10}\frac{R^{2}}{MSE}$$

Here, *MSE* is the difference in each pixel between two images, and R, the maximum value of the corresponding image, is used when evaluating the image loss information.

Additionally, the performance evaluation of the proposed multimodal object detection model, including DN-RTD, was based on the mean Average Precision (mAP), which expresses the area below the precision–recall (PR) curve and shows how reliable a model is against the detected object. Precision is the ratio of the correctly detected results among those detected and is given as TP/(TP + FP). A recall is the ratio of the correctly detected object among those that should be detected and is given as TP/(TP + FN). Here, TP, FP, and FN denote true positive, false positive, and false negative, which means that objects that should be detected were not detected.

### 5.1. Evaluations on Image Dehazing

For the CNN training to estimate the atmospheric scattering coefficient at a specific scope with single images, data labeled with the scattering coefficient are required. Authors of [21,33,34] generated the simulated hazed image data using an atmospheric scattering model and used it for model training. Similarly, we generated simulated hazed images using arbitrary scattering coefficients and an atmospheric scattering model. The scattering coefficients were categorized into four levels, each generating 577 test images. Table 2 summarizes the identification accuracy, which is the CNN-based environment identification performance index by haze level. The identification accuracy was at 87.12% on average, verifying that the model showed stable performance on the weather environment identification by haze levels.

**Figure 5:**
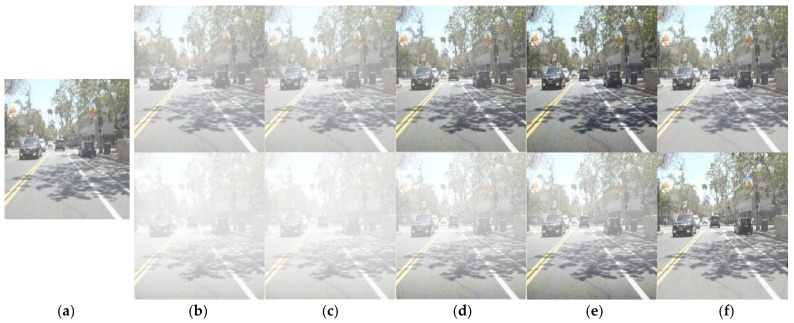
Example of dehazing performance comparison by model (top: light haze, bottom: dense haze); (**a**) original, (**b**) hazed image, (**c**–**f**) dehazed image by [23,24,33], and DN-RTD, respectively.

Using the atmospheric scattering coefficients estimated from the above, we compared the performance of our proposed dehazing model, DN-RTD, to those of existing image dehazing models, the result of which is summarized in Table 3 with the process examples shown in Figure 5. Under the light haze level, our proposed model showed higher dehazing performance than [24], which showed the highest image quality improvement among the existing models. For the dense haze level, our proposed model demonstrated substantially higher dehazing performance than all other models. The visual quality of the proposed model also showed higher dehazing performance at all haze levels.

### 5.2. Comparative Evaluations of Detection under Hazy Conditions

To demonstrate the superiority of the proposed deep multi-mode object detection model (YOLO^RT^ + DN-RTD) including DN-RTD for image dehazing, object detection performance evaluation was carried out with various procedures. A comparison test was performed on the proposed architecture according to the level of visibility, and a comparative evaluation was also conducted with the object detection model, which fused with various conventional haze removal models and YOLO.

Table 4 shows a comparison of the object detection performance between other state-of-the-art detection models and the proposed YOLO^RT^ + DN-RTD model. Under the light haze level with a relatively low effect of haze, YOLO^RT^, which is a late fusion model of RGB and thermal images, showed improved detection performance of AP by approximately 6% or more than the RGB image-based model YOLO^R^ and thermal image-based model YOLO^T^. For the dense haze level, the detection performance only by RGB images dropped significantly because of the considerable effect of haze. This also affected the multimodal model YOLO^RT^, dropping the AP by approximately 4% or more in the light haze level. However, the performance increased by about 3% more than thermal image single sensor detection model YOLO^T^ due to the fusion with thermal images, verifying that detection performance can be improved by multimodal sensor fusion. The proposed YOLO^RT^ + DN-RTD showed improvement in object detection by up to 8% above the existing image dehazing models and YOLO^R^ under the light haze level, and by about 1.4% above YOLO^RT^. The dehazing performance of existing image dehazing models under the dense haze level decreased, causing a huge drop in detection performance. However, the proposed YOLO^RT^ + DN-RTD model removed haze according to each haze level. Thus, even in a dense haze environment like fog, its AP improved by 22% or higher above YOLO^R^+ [33] and by about 3% to YOLO^RT^. Therefore, the proposed object detection model improved the detection performance using RGB images through appropriate dehazing and showed stable detection potential under poor weather conditions like fog due to its stable image dehazing performance as opposed to existing dehazing models. The proposed architecture performed equivalent to or faster execution speed than the object detection models combining the conventional haze removal models and YOLO. However, since the total processing time from haze removal to object detection is 686.99 ms, it seems that it will be rather difficult to process without interruption in real time. This is because real-time processing is possible at 27.89 ms based on the object detection time, but 659.1 ms, which is most of the total processing time, is required in the haze removal process like other haze removal techniques. Therefore, further studies are planned in the future to accelerate the haze removal process to increase its real-time potential.

**Figure 6:**
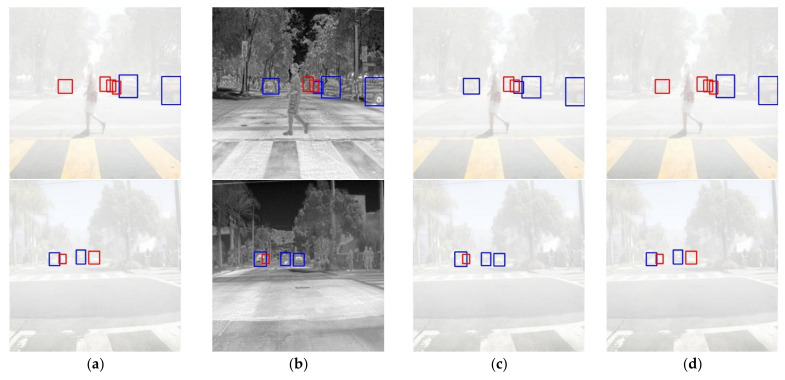

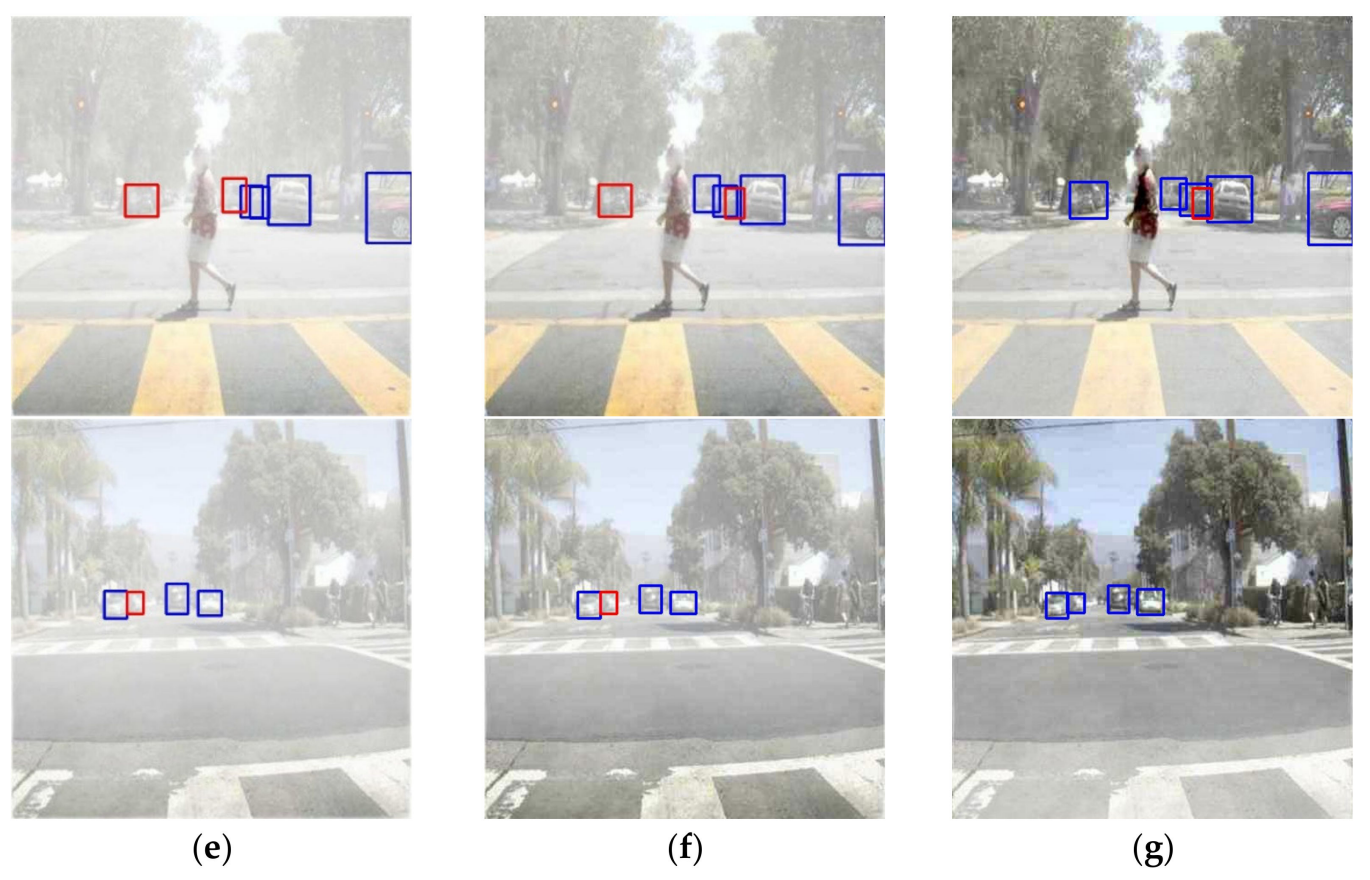
Examples of vehicle detection results via model under reduced visibility (Red box—Missed detection, Blue box—Correct detection); (**a**) YOLO^R^, (**b**) YOLO^T^, (**c**) YOLO^RT^, (**d**) YOLO^R^ + [33], (**e**) YOLO^R^ + [24], (**f**) YOLO^R^ + [23], (**g**) YOLO^RT^ + DN-RTD.

Figure 6 shows examples of vehicle detection by model under reduced visibility with a dense haze level. In particular, Figure 6a shows that relatively distant objects were not detected at all due to haze. Figure 6b shows that regardless of the haze level, most objects could be detected using clear boundary lines, but for objects near tree branches, the boundary lines of the branches and those of the moving targets were mixed so that the objects could not be detected, leading to missed detection. Therefore, it was confirmed that the proposed multimodal object detection model improved the detection performance using the fusion of the thermal sensor and allowed complementary detection of objects that the other sensors could not find. Furthermore, many more objects could be detected with superior image dehazing performance of the proposed model to those of existing image dehazing algorithms under the dense haze level.

## 6. Conclusions

Moving target detection is a crucial task in intelligent personal mobility and autonomous driving. While the detection techniques of moving targets using camera sensors have witnessed a high level of accuracy and rapid execution speed due to the development of deep learning, its performance considerably drops under poor weather conditions. Haze, one of the most common weather conditions where poor visibility results from vapors or dirt in the atmosphere, degrades the performance of vision-based applications in autonomous driving. Thus, the development of an image dehazing model for stable moving target detection is required. Deep learning-based dehazing methods, which estimate a transmission map using neural networks, are easy to implement, have faster processing speed than previous techniques, and have acquired versatility using big data. However, when estimating a transmission map, they infer detailed parameters of an atmospheric scattering model for end-to-end training, leading to error accumulation and degraded accuracy since they fail to consider the depth, which is the most important parameter. Therefore, we proposed an image restoration model that improves detection performance by identifying surrounding environment from images, detecting the haze level, extracting depth information from a single image, and removing haze for stable object detection under reduced visibility due to haze. Additionally, we proposed a multimodal object detection scheme that improves detection performance through the late fusion of the restored RGB and thermal images. The proposed dehazing model showed improved dehazing performance by up to 10% or more compared with existing CNN-based image dehazing algorithms and demonstrated the potential of dehazing under fog. Finally, the proposed model showed improved detection performance by up to 22% or more to the model that combined the existing CNN-based dehazing technique and YOLO, verifying the validity of the proposed model.

## Figures and Tables

**Table 1:** Estimated atmospheric scattering coefficients by haze level.

CNN Output (G)	Haze Level	Atmospheric Scattering Coefficient (β)	Estimated Atmospheric Scattering Coefficient $\left(\bar{\beta }\right)$
1	Haze-free	0~0.50	0.25
2	Light haze	0.51~1.00	0.75
3	Moderate haze	1.01~1.50	1.25
4	Dense haze	1.51~2.30	2.00

**Table 2:** Environment identification performance by haze level.

Haze Level	Haze-Free	Light Haze	Moderate Haze	Dense Haze
Identification Accuracy	99.65%	78.16%	81.10%	89.60%

**Table 3:** Environment identification performance by haze level.

Model	PSNR		SSIM	
	Light Haze	Dense Haze	Light Haze	Dense Haze
[33]	18.14	13.24	0.88	0.66
[24]	22.63	15.93	0.92	0.76
[23]	18.11	17.96	0.90	0.83
DN-RTD	28.69	23.56	0.96	0.89

**Table 4:** Vehicle detection performance comparison by model according to the haze level.

Models	Light Haze					Dense Haze					Running Time (ms)
	AP	TP	FP	Precision	Recall	AP	TP	FP	Precision	Recall	
YOLO^R^	78.75	1615	39	0.97	0.79	61.55	1264	30	0.97	0.61	27.82
YOLO^T^	78.60	1626	182	0.90	0.79	78.60	1626	182	0.90	0.79	27.82
YOLO^RT^	85.22	1767	241	0.88	0.86	81.11	1683	238	0.87	0.82	27.89
YOLO^R^ + [33]	78.75	1615	39	0.97	0.79	61.60	1265	30	0.97	0.61	657.82
YOLO^R^ + [24]	81.35	1668	27	0.98	0.81	66.99	1374	18	0.99	0.67	1627.82
YOLO^R^ + [23]	83.41	1711	55	0.97	0.83	71.55	1468	32	0.98	0.71	737.82
YOLO^RT^ + DN-RTD	86.69	1800	279	0.86	0.88	84.02	1747	269	0.86	0.85	686.99

## Data Availability

The data presented in this study are available on request from the corresponding author.
